# Flexible Endoscopy Versus Rigid Endoscopy or Surgery for the Management of Zenker’s Diverticulum: A Systematic Review and Meta-Analysis

**DOI:** 10.7759/cureus.43021

**Published:** 2023-08-06

**Authors:** Diego P Cadena Aguirre, Diogo Turiani Hourneaux de Moura, Bruno Hirsch, Guilherme Henrique Peixoto de Oliveira, Angelo So Taa Kum, Sultan Mahmood, Wanderley Marques Bernardo, Neil R Sharma, Eduardo Guimarães De Moura

**Affiliations:** 1 Serviço de Endoscopia Gastrointestinal do Departamento de Gastroenterologia, Hospital das Clínicas da Faculdade de Medicina da Universidade de São Paulo, São Paulo, BRA; 2 Gastroenterology, Marshfield Clinic, Marshfield, USA; 3 Division of Interventional Oncology and Surgical Endoscopy (IOSE), Parkview Health, Fort Wayne, USA

**Keywords:** surgery, endoscopy, systematic review, meta-analysis, cricopharyngeal myotomy, zenker diverticulum

## Abstract

Zenker's diverticulum treatment options range from endoscopic rigid or flexible procedures to surgery. There are limited studies available comparing these techniques. Frequently, the choice of treatment depends on the physician's preference or experience, as well as the institution's resources and capacity. Therefore, this study aims to define the best approach based on the highest efficacy and the lowest severe adverse events. In accordance with Preferred Reporting Items for Systematic Reviews and Meta-Analyses (PRISMA) guidelines, a comprehensive search was performed. Only comparative studies were included, analyzing flexible endoscopy versus rigid endoscopy or surgery. The outcomes analyzed were clinical and technical success, severe adverse events, length of stay, and duration of the procedure. Analysis was performed using Review Manager 5.4.1 (RevMan 5.4, The Cochrane Collaboration, The Nordic Cochrane Centre, Copenhagen, Denmark). Eight retrospective cohort studies met the inclusion criteria. A total of 1281 patients were identified, 492 underwent flexible endoscopy, 453 underwent rigid endoscopy, and 336 underwent surgery. There was no difference in clinical success [risk difference (RD), 0.07 (95% CI -0.05 to 0.19%); P = 0.26], technical success [RD, 0.07 (95% CI -0.03 to 0.16); P = 0.18], severe adverse events [RD, -0.03 (95% CI -0.13 to 0.07; P = 0.052), perforation [RD, 0.07 (95% CI -0.04 to 0.19); P = 0.22] or procedure time [mean difference (MD), - 10.03 (95% CI -26.93 to 6.88); P = 0.24). There was lower length of stay with flexible endoscopy compared to the other approaches [MD, -1.98 (95% CI -3.56 to -0.40); P = 0.001]. Based on the current evidence, the three main techniques are effective for the treatment of Zenker’s diverticulum. Although there was no significant difference in the safety of each technique in this meta-analysis, this result should be interpreted cautiously due to the limited data and the risk of vies between the techniques, considering that the results tend to favor flexible endoscopy, mainly explained by the newer and safer devices. Length of stay is lower with flexible endoscopy versus the other techniques, which can be beneficial considering the geriatric populations where Zenker’s diverticulum mainly occurs.

## Introduction and background

Zenker's diverticulum (ZD) is a false diverticulum located between the inferior constrictor muscles of the pharynx and the cricopharyngeus muscle. It is a rare disease that primarily affects elderly people, generally over 70 years of age. Patients with ZD present with symptoms such as dysphagia, regurgitation, and halitosis, and in severe cases, it can lead to complications like aspiration pneumonia or malnutrition [1-3].

Currently, there are three therapeutic options: flexible endoscopy (FE), rigid endoscopic (RE) treatment, and surgery. FE septotomy, developed for the first time in 1982 by Professor Dr. Sakai, is the technique with the most innovations in recent years, including the development of high-definition equipment, the use of CO_2_, a diverticuloscope for ZD exposure, a better cutting and coagulation system, as well as safer and more precise endoscopic knives [4-5].

Although several studies have been published, there is no consensus on the gold standard treatment. The aim of this systematic review and meta-analysis was to define the best approach for ZD treatment based on the highest efficacy and low rates of severe adverse events (SAEs).

## Review

Materials and methods

Protocol and Registration

The present meta-analysis was carried out in accordance with the Preferred Reporting Items for Systematic Reviews and Meta-Analysis (PRISMA) guideline [6,7]. The study protocol was registered in the International Prospective Register of Systematic Reviews (PROSPERO) under the code CRD42023402160.

Eligibility Criteria

Only comparative studies were included, analyzing either FE versus RE or surgery. The studies were limited to the English, Portuguese, and Spanish languages, without a year restriction. The population of interest was all patients aged 18 years or older diagnosed with ZD. Studies with missing data were excluded. Eligibility was determined by reviewing the titles and abstracts of all potential studies to be included; two authors independently screened each review.

Information Sources and Search Strategy

The PICO question was formulated before conducting the search on the main databases to establish the relevance of the articles in addressing the purpose of the present study. A comprehensive search was performed using electronic medical databases (MEDLINE, Embase, ClinicalTrials.gov, LILACS, Cochrane Database) and Google Scholar.

The medical subject headings (MeSH) terms were used aiming to select comparative studies with the highest quality possible. The following MeSH terms were used: (Diverticulum, Zenker OR Pharyngoesophageal Diverticulum OR Pharyngeal Diverticula OR Pharyngoesophageal Pulsion Diverticula OR Esophageal Pharyngeal Diverticulum OR Esophagopharyngeal Diverticulum) AND (Endoscopy OR Endoscopic). The last search was run on March 22, 2023, with the original MeSH terms used to create the initial database.

Study Selection and Data Collection Process

Demographic characteristics, the number of included patients, treatment method, clinical success, technical success, SAE, perforation, LOS, and procedure time were extracted by the two reviewers independently. Disagreements in the process of data extraction were resolved by discussion and consensus. The data of interest were collected and organized in a spreadsheet.

Data Items

The primary outcome was clinical success, defined as remission of dysphagia. The secondary outcomes were technical success (defined as successful completion of all procedural steps), SAE (defined as Clavien Dindo Score >2), perforation, length of stay (LOS) (days), and length of the procedure (minutes). Missing data were not tabulated.

Risk of Bias and Quality of Evidence

The Risk of Bias in Non-randomized Studies (ROBINS-I) was employed for internal validation and to assess the risk of bias in comparative observational studies.

Effect Measure and Synthesis of the Methods

The clinical data of the patients of each study were recorded in a spreadsheet. For all data analyses, a 95% confidence interval (CI) and P < 0.05 were used to define them as statistically significant. Discrete variables were analyzed using absolute numbers, and continuous variables were analyzed using means and standard deviation (SD). For continuous outcomes, the inverse variance test was used, and for dichotomous endpoints, the difference was calculated by the risk difference (RD), using the Cochrane Mantel-Haenszel test. The heterogeneity of the studies was analyzed with I2, based on the Higgins and Thompson method [8]. Subgroup analysis comparing FE versus RE or FE versus surgery was conducted to explore possible causes of heterogeneity. The random effect model was used in all the analyses. The software Review Manager software version 5.4.1 (RevMan 5.4, The Cochrane Collaboration, The Nordic Cochrane Centre, Copenhagen, Denmark) was used for metanalysis.

Certainty Assessment

The certainty of the evidence for the outcomes was assessed using Grades of Recommendation, Assessment, Development, and Evaluation (GRADE) and the GRADEpro software (McMaster University and Evidence Prime, Inc., Hamilton, Canada).

Results

Study Selection

**Figure 1:**
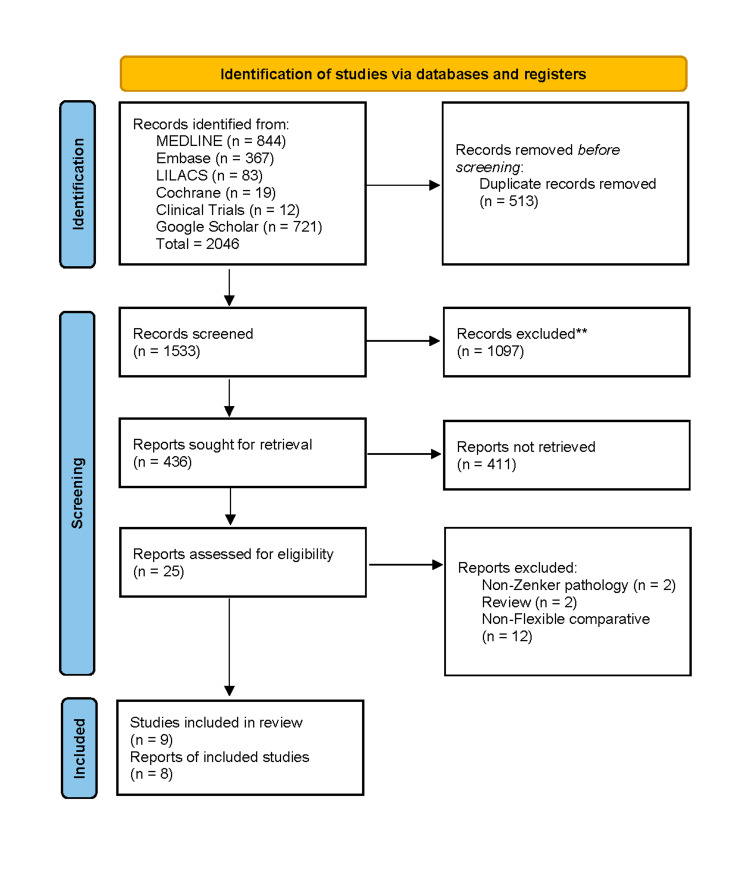
PRISMA flow diagram.

The search strategy identified 2046 articles: 844 studies were identified in the MEDLINE database, 367 in Embase, 83 in LILACS, 19 in Cochrane Library, 12 in Clinical Trials, and 721 in Google Scholar. After removing duplicate studies and applying the eligibility criteria, 25 articles were chosen for full-text review. Finally, nine articles satisfied all eligibility criteria and were included in the meta-analysis. A PRISMA flow chart of search results is shown in Figure 1.

Characteristics of the Studies

Eight retrospective cohort studies were included. A total of 1281 patients were analyzed: 492 underwent FE and 789 underwent non-flexible endoscopy. The last group was divided into RE and surgery, with 453 and 336 patients, respectively. The main outcomes of each study are summarized in Table 1.

**Table 1:** Summary of the characteristics of the studies that met the inclusion criteria. FE, flexible endoscopy; RE, rigid endoscopy; SD, standard deviation; NR, not reported

Study	No. of patients				Clinical success			Technical success		Severe adverse events			Perforation		Mean length of stay (days)			Mean procedure time (min)	
	Total	FE	RE	Surgery	FE	RE	Surgery	FE	RE	FE	RE	Surgery	FE	RE	FE (SD)	RE (SD)	Surgery (SD)	FE (SD)	RE (SD)
Dolan, 2021 [9]	102	51	51	NR	39/51	23/51	NR	16/22	29/41	NR	NR	NR	9/51	1/51	0.9 (2.2)	3.5 (6.1)	NR	83 (33)	73.5 (9.7)
Calavas, 2021 [10]	106	49	57	NR	37/39	40/43	10/13	NR	NR	NR	NR	NR	3/53	6/58	NR	NR	NR	40 (19)	27 (16)
Al Ghamdi, 2021 [11]	245	205	40	NR	167/185	33/37	NR	195/205	35/40	NR	NR	NR	22/205	12/30	1.47 (0.97)	4.94 (14.75)	NR	33.72 (22.34)	54.03 (22.45)
Runge, 2020 [12]	240	60	NR	180	52/60	NR	162/180	NR	NR	0/60	NR	14/180	NR	NR	1.2 (2.5)	NR	3.4 (4.1)	NR	NR
Jones, 2014 [13]	52	11	8	33	NR	NR	NR	NR	NR	0/11	1/8	3/33	NR	NR	NR	NR	NR	NR	NR
Wander, 2014 [14]	54	18	NR	36	NR	NR	NR	NR	NR	2/18	-	7/36	NR	NR	0.44 (0.25)	NR	3.31 (0.3)	NR	NR
Repici, 2011 [15]	58	28	30	NR	NR	NR	NR	NR	NR	1/28	2/30	-	0/28	1/30	NR	NR	NR	60 (15)	100 (20)
Wallerius, 2023 [16]	424	70	267	87	NR	NR	NR	NR	NR	11/70	12/267	9/87	10/70	11/267	1.3 (2.74)	1.1 (1.13)	1.2 (1.50)	33.2 (15.68)	45.86 (20.88)

Clinical Success

**Figure 2:**
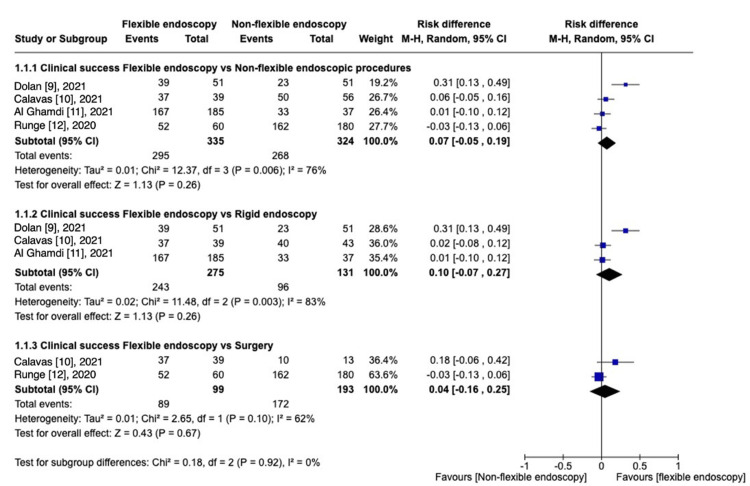
Clinical success: FE vs. other procedures.

Four studies [9-12] assessed this outcome. FE was compared with non-flexible procedures, including RE and surgery, and RD was calculated [RD, 0.07 (95% CI -0.05 to 0.19%); I^2^ = 76%; P = 0.26]. Subgroup analyses were performed, the first one comparing FE to RE [9-11] [RD, 0.10 (95% CI -0.07 to 0.27%); I^2^ = 83%; P = 0.26], and the second comparative subgroup [10,12] between FE versus surgery reported [RD, 0.04 (95% CI -0.16 to 0.25); I^2^ = 62%; P = 0.67] (Figure 2).

Technical Success

**Figure 3:**
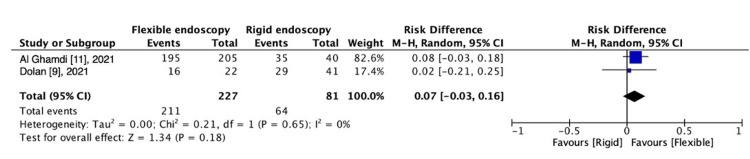
Technical success: RE vs. FE.

Two studies evaluated technical success [9,11] comparing FE versus RE; a non-significant difference was observed [RD, 0.07 (95% CI -0.03 to 0.16); I^2^ = 0%; P = 0.18] (Figure 3).

Severe Edverse Events

**Figure 4:**
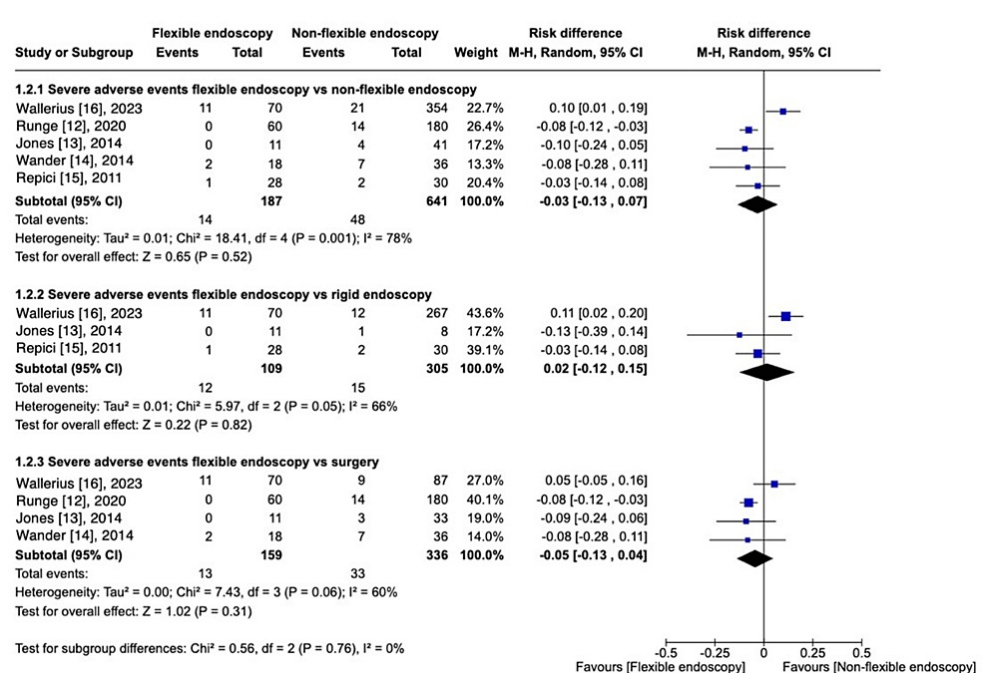
Severe adverse events: FE vs. Non-flexible procedures.

Five articles [12-16] reported SAE, totaling 828 patients. There is no significant difference in the rate of SAE events compared to non-flexible procedures, including RE and surgery [RD, -0.03 (95% CI -0.13 to 0.07); I^2 ^= 78%; P = 0.052) (Figure 4). Subgroup analysis comparing FE versus RE [13,15] presented no significant difference [RD, 0.02 (95% CI -0.12 to 0.15); I^2^ = 66%; P = 0.82). When comparing FE and surgery [9,13,14], there was not a statistically significant difference of SAE either [RD, -0.05 (95% CI -0.13 to 0.04); I^2 ^= 60%; P = 0.31).

Perforation

**Figure 5:**
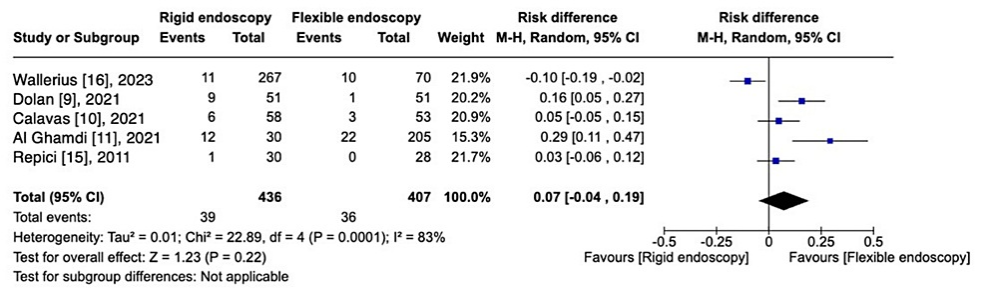
Perforation: RE vs. FE.

Five studies [9-11,15,16] reported perforation rate, totaling 75 perforation cases in 843 patients analyzed. There is no significant difference on the risk of perforation between FE and RE [RD, 0.07 (95% CI -0.04 to 0.19); I^2 ^= 83%; P = 0.22] (Figure 5).

Length of Stay

**Figure 6:**
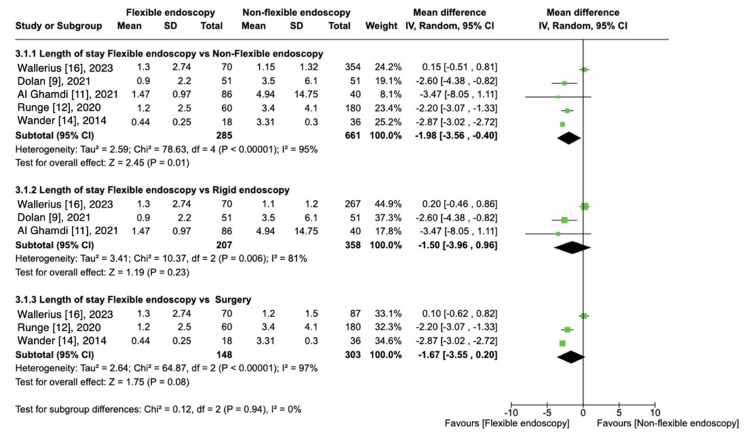
Length of stay: FE vs. other procedures.

Five studies reported LOS [9,11,12,14,16] totaling 946 patients. There was a statistically significant difference favoring FE versus non-flexible endoscopy approach [MD, -1.98 (95% CI -3.56 to -0.40); I^2 ^= 95%; P = 0.001]. There was no difference in LOS with FE versus RE [9,11,16] once this subgroup was analyzed [MD, -1.50 (95% CI -3.96 to 0.96); I^2^ = 81%; P = 0.23]. The second subgroup comparing FE vs surgery [12,14,16], showed no difference in LOS either [MD -1.67 (95% CI -3.55 to 0.20); I^2 ^= 97%; P = 0.08] (Figure 6).

Procedure Time

**Figure 7:**
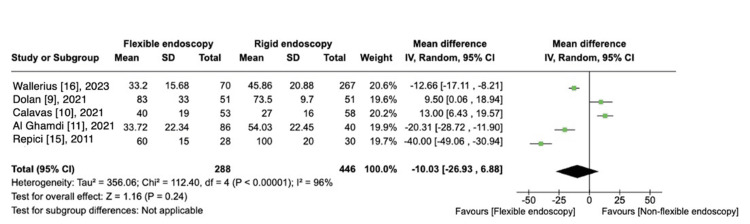
Procedure time: FE vs. RE.

Five studies reported procedure time, comparing FE versus RE [9-11,15,16], totaling 734 patients. There was no difference on the procedure time comparing both techniques [MD, -10.03 (95% CI -26.93 to 6.88); I^2^ = 96%; P = 0.24) (Figure 7).

Risk of Bias and Quality of Studies

**Figure 8:**
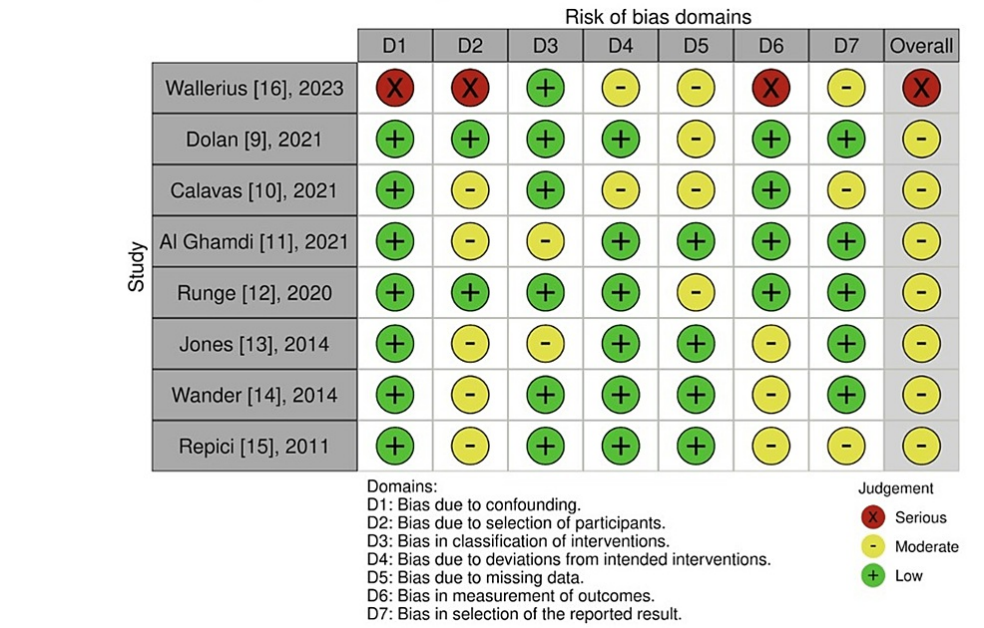
Risk of bias (ROBINS-I tool).

The ROBINS-I (Figure 8) considered the overall risk of bias moderate.

The quality of evidence, as assessed by the GRADE methodology, is demonstrated in Table 2.

**Table 2:** Quality of evidence assessed by the Grading of Recommendations Assessment, Development, and Evaluation criteria (GRADE). CI: confidence interval; MD: mean difference; OR: odds ratio ^a^Different scores have been used to define dysphagia. ^b^Heterogeneity more than 50%. ^c^Wide CI. ^d^Different scores have been used to define severe adverse outcomes. ^e^Not directness of the study. ^f^Heterogeneity more than 90%.

Certainty assessment							№ of patients		Effect		Certainty	Importance
№ of studies	Study design	Risk of bias	Inconsistency	Indirectness	Imprecision	Other considerations	Flexible endoscopy	Nonflexible approach	Relative (95% CI)	Absolute (95% CI)		
Clinical success Flexible endoscopy versus surgery												
2	Observational studies	Serious ^a^	Serious ^b^	Not serious	Serious ^c^	All plausible residual confounding would suggest a spurious effect, while no effect was observed dose-response gradient	89/99 (89.9%)	172/193 (89.1%)	OR 1.66 (0.23 to 11.88)	40 more per 1,000 (from 238 fewer to 99 more)	⨁⨁⨁◯ Moderate	Important
Clinical success flexible endoscopy versus rigid endoscopy												
3	Observational studies	Serious ^a^	Serious ^b^	Not serious	Serious ^c^	Strong association dose-response gradient	243/275 (88.4%)	96/131 (73.3%)	OR 0.10 (-0.07 to 0.27)	100 fewer per 1,000 (from 270 fewer to 70 more)	⨁⨁⨁◯ Moderate	Important
Technical success flexible endoscopy versus surgery												
2	Observational studies	Not serious	Not serious	Not serious	Serious ^c^	None	211/227 (93.0%)	64/81 (79.0%)	OR 0.07 (-0.03 to 0.16)	70 fewer per 1,000 (from 160 fewer to 30 more)	⨁⨁⨁◯ Moderate	Important
Severe adverse events flexible endoscopy versus rigid endoscopy												
3	Observational studies	Serious ^d^	Not serious	Not serious	Serious ^c^	Strong association of all plausible residual confounding would reduce the demonstrated effect of a dose-response gradient	12/109 (11.0%)	15/305 (4.91%)	OR 0.02 (-0.12 to 0.15)	50 more per 1,000 (from 60 fewer to 150 more)	⨁⨁⨁⨁ High	Important
Severe adverse events flexible endoscopy versus surgery												
4	Observational studies	Serious ^b^	Not serious	Not serious	Not serious	None	13/159 (8.17%)	33/336 (9.82%)	OR -0.05 (-0.13 to 0.04)	80 more per 1,000 (from 40 more to 120 more)	⨁⨁⨁◯ Moderate	Important
Perforation flexible endoscopy versus rigid endoscopy												
5	Observational studies	Not serious	Serious ^b^	Not serious	Not serious	Strong association	39/436 (8.94%)	36/407 (8.85%)	OR 0.07 (-0.04 to 0.19)	120 fewer per 1,000 (from 230 fewer to 0 fewer)	⨁⨁⨁⨁ High	Important
Length of stay Flexible endoscopy versus Rigid endoscopy												
3	Observational studies	Not serious	Not serious	Serious ^e^	Not serious	None	207	358	-	MD 1.50 lower (3.96 lower to 0.96 higher)	⨁⨁⨁◯ Moderate	Important
Length of stay flexible endoscopy versus surgery												
3	Observational studies	Not serious	Serious ^a^	Serious ^c^	Not serious	Dose-response gradient	148	303	-	MD 1.67 lower (3.55 lower to 0.20 higher)	⨁⨁⨁◯ Moderate	Important
Length of the procedure (minutes) flexible endoscopy versus rigid endoscopy												
5	Observational studies	Not serious	Very serious ^f^	Serious ^e^	Serious ^c^	Strong association of all plausible residual confounding would reduce the demonstrated effect of a dose-response gradient	288	446	-	MD 10.03 lower (26.93 lower to 6.88 higher)	⨁⨁⨁◯ Moderate	Important

Discussion

ZD is a disease that was described by Zenker in 1877. Even though its treatment began more than a century ago, there is no consensus among surgeons and endoscopists recommending the best therapeutic option. This is the first meta-analysis only using comparative studies between FE versus RE or surgery. Unlike previous meta-analyses that used non-comparative studies between FE and the other techniques [17-26].

To date, there is no gold standard for ZD treatment. In the present study, there was no significant difference when comparing clinical success between FE, RE, or surgery in terms of resolving dysphagia. This outcome depends on the capacity to perform a complete septotomy, communicating the diverticular sac with the esophageal lumen, and all three techniques are capable of achieving it. However, each technique has its own characteristics that must be considered when choosing the most effective method. These differences must be recognized, as they can lead to varying rates of clinical success or failure, each with different adverse event rates. Contrary to the notion that the chosen technique should be solely based on the physician's preference [2], understanding these distinctions is crucial in making an informed decision for ZD treatment.

FE has many advancements, such as new high-definition scopes that can show more clearly the buccopharyngeal fascia, which is the landmark to ensure a complete septotomy. This anatomical reference is difficult to evidence with RE or surgery. This advantage can theoretically increase the chances of clinical success and decrease the risk of recurrence or persistence of symptoms, due to easier recognition of this anatomical reference [4]. Also, the combination of FE and diverticuloscope might potentially improve safety during diverticulotomy, although this outcome has not been reproduced in other studies [27].

Two crucial factors limit the efficacy of RE diverticulotomy: insufficient cervical hyperextension and septal thickness. Wilsen described a case series, where 35.3% of stapler septotomies failed due to these two factors. Insufficient cervical hyperextension is common in the geriatric population (23.5%), making septotomy with a stapler more difficult. In comparison, FE does not require cervical hyperextension for septotomy, so FE can be an excellent choice for patients with limited cervical extension. The second cause of failure (11.8%) was a very thick septum, making it difficult to close the stapler and perform an incomplete septotomy [28]. The FE can perform a complete septotomy without considering the thickness of the septum, as the endoscope can direct the knife through its entire thickness. Other factors that can hinder technical success with RE are prominent front teeth, dental implants, lockjaw, and jaw stiffness [29].

Technical success is defined as the successful completion of all procedural steps with an adequate complete cricomyotomy. Our meta-analysis showed no difference between FE and RE in terms of technical success [9,11]. A small fraction of patients in both groups had to undergo surgery to complete the myotomy or due to an adverse event. Intraprocedural perforation with difficult endoscopic closure with clips is one of the possible scenarios. Al Ghamdi et al. have reported the use of metallic stents in four patients. The available literature recommends closure with through-the-scope clips for defects <1 cm or considering over-the-scope clips for defects <2 cm, and it also supports the limited use of partially covered or fully covered metal stents [30]. Perforation closure with RE is technically demanding, so an alternative technique must be considered (FE or surgery). Meanwhile, if perforation occurs in FE, most of them can be treated with the same technique, using clips, stents, or endoscopic vacuum [30,31].

There is no significant difference in SAE between FE, RE, and surgery. When comparing the risk of perforation between FE and RE, no significant difference was observed either. Of note, only one study used in this meta-analysis [16] showed a higher safety profile with non-flexible endoscopy. This trial could have a risk of selection bias, as the patients submitted to FE were older than the other groups. In the same study, 80% of the perforations and adverse severe events with FE occurred during the first four years of the study period. The same authors of this study attribute these results to the learning curve needed, more rudimentary cutting needle knives, and operator technique in the first half of the study [16].

At this point, it is crucial to highlight the rapid development of new endoscopic technologies, such as enhanced imaging through high-definition scopes, facilitating safe therapeutic interventions. Also, current scopes are smaller and more flexible than their historic counterparts, permitting safer maneuverability. The use of wide-angle scopes and different kinds of caps are novel innovations that should be considered when selecting the safest approach to ZD [32].

There are many options for knives, each with different configurations to mitigate the side effects of thermal injury during the septotomy. Additionally, the use of scissor-type ESD knives has been described, mainly due to their advantages of grasping, retracting, and coagulating [33].

Some advocates suggest using hemostatic clips in FE to prevent bleeding and delayed perforation due to thermal injury, which can help reduce the risk of perforation with FE [10]. To further reduce the risk of perforation, which generally occurs at the bottom of the septum, some authors advocate leaving a small rim of septum at the base [11], but theoretically, this may lead to lower clinical success and a higher risk of recurrence.

Another adverse event not analyzed in this meta-analysis but important when deciding on the treatment is the evaluation of the oral cavity. A meta-analysis found that 25.8% of patients in the laser septotomy group and 27.9% in the stapler group (RE techniques) presented dental trauma [34]. If the oral cavity is not favorable for performing an RE, then FE is a good option.

General anesthesia is mandatory in RE and surgery, although it is also recommended in therapeutic endoscopy. One study reported that only 65% of ZD procedures using FE used general anesthesia. If, after pre-anesthesia evaluation, a high perioperative risk with general anesthesia or a high likelihood of orotracheal intubation failure is found, FE under deep sedation can be considered [11].

LOS is lower in FE than in RE or surgery. Individual studies also report less time for liquid or food resumption after FE, while surgery has the highest LOS time followed by RE. Most FE procedures are routinely performed as outpatient procedures and are less traumatic compared to RE or surgery [10]. It is important to consider LOS, as the geriatric population is susceptible to healthcare-associated infections (HAI). A study reported that age, when combined with other comorbidities such as cardiovascular disease, neurological deterioration, or diabetes mellitus, increases the risk of HAI [35]. A shorter hospital stay could reduce the risk of HAI, so this benefit of FE over other procedures should be considered.

Procedure time was only compared between FE and RE, showing no differences in procedure time. This outcome is difficult to ascertain due to the heterogeneity of the data, as there are different kinds of procedures that can be done with FE, such as Z-POEM, peroral endoscopic septotomy (POES), septotomy, and more. Similarly, RE can be performed with a CO_2_ laser or stapler [9-11,15,16], which explains the different mean times of the procedures and the high heterogeneity of this outcome.

This systematic review could not analyze recurrence due to the absence of follow-up in the selected studies. Previous meta-analyses have reported recurrence, defined as the need for a second procedure due to the persistence of symptoms, showing that surgery had a lower risk of recurrence than RE [24]. The size of the diverticulum has been proposed to be a factor for recurrence. Based on this, surgery used to be recommended if the size was larger than 4 cm. In a larger diverticulum, the preference was to perform a diverticulectomy or diverticulopexy concomitantly or not with cricopharyngeus myotomy. However, contrary to expectations, a meta-analysis showed that recurrence was higher in the group with a small-size diverticulum than in the larger size group (18% vs 8%, respectively) [20].

Only one cost-effectiveness study comparing RE vs surgery has been conducted. It is important to consider the effectiveness as well as the economic impact for the treatment of ZD. Eleven studies were used to create a probabilistic sensitivity analysis, where surgery had a lower risk of recurrence than FE (5.3% vs 7.6%), but surgery had a higher complication rate than FE (12% vs 6%). They concluded that endoscopic surgery is the more cost-effective strategy compared to open surgery. This benefit should be considered when deciding the ZD approach. For the moment, there is no cost-effectiveness study with the FE technique, but the same advantages of RE over surgery would lead one to think that FE is also better than surgery in terms of economic impact.

The European Society of Gastrointestinal Endoscopy (ESGE) is the only guideline to make any recommendation about the ideal technique to treat ZD. There establishes the FE as the first choice of treatment, regardless of the diverticulum’s size, and current evidence shows a good response with repeated sessions of FE in case of recurrence [36]. But this is a weak recommendation and is based on low-quality evidence. This meta-analysis can be used to guide further recommendations on ZD treatment. Additional randomized clinical trials are needed to better answer these questions, but current evidence shows a clear benefit with FE in terms of LOS over the other techniques while providing a similar clinical success.

Our study has some limitations. All included studies were retrospective, and in some cases, the decision to allocate patients to each group was not standardized, which may lead to selection bias. For this reason, the overall risk of bias is considered moderate. The absence of follow-up could bias the long-term outcome and recurrence. However, this meta-analysis helps to define the most appropriate treatment for ZD based on the best current evidence, comparing the three main groups of techniques available at the moment: FE, RE, and surgery. Minimally invasive endoscopic techniques have unique advantages; mainly, SAE can be managed by the same endoscopically technique, and there is a lower demand for medical health care assistance as it could be done in an ambulatory setting.

In summary, this meta-analysis provides enough evidence to define that FE is as effective and safe as surgery or RE. Individual studies show an important difference in SAE, which favors FE over the other techniques. Additionally, FE provides outpatient care associated with faster liquids and food resumption. The lower cost and more rapid recovery for patients are important practical considerations. The results of this study can be used in the decision-making process with patients with ZD based on local expertise and available infrastructure to provide the most effective technique with fewer adverse events.

## Conclusions

Despite more than a century since the first treatment for ZD, this is the first meta-analysis using only comparative studies for the treatment of ZD, aiming to elucidate the best treatment option among surgery, RE, or FE. Our meta-analysis supports the current recommendation that the three available techniques are equally effective in the remission of dysphagia.

Another important aspect to consider before undergoing any treatment is the safety of the technique. This study did not demonstrate any significant difference in terms of SAE. The FE approach has experienced important development in the last decade, with safer endoscopes offering better image quality and new endoscopic scalpels now available.

Finally, the choice of procedure must consider the experience of the physician, the available infrastructure, and the limitations of the patient, such as limited neck extension and clinical comorbidities. FE shows a shorter LOS, facilitating outpatient treatment and reducing exposure to HCI in the geriatric population with ZD.
